# A Formulary for the Preparation and Medical Administration of Certain New Remedies

**Published:** 1836-07

**Authors:** 


Art. XI.—*Formulaire pour la Préparation et l'Emploi de plusieurs nouveaux Médicaments.* Par F. Magendie. Huitième édition, revue et augmentée: à Paris. 1835. 12mo. pp. 438.

*A Formulary for the Preparation and Medical Administration of certain new Remedies.* Translated from the French of M. Magendie, with annotations and additional articles, by James Manby Gully, m.d. London, 1835. 12mo. pp. 216.

The principal substances which have been added in the present edition of this celebrated formulary are, codeine, narceine, meconine, prussic ether, lactic acid, and mannite: we will proceed to give a short account of them.

*New principles discovered in opium.—Narceine* was discovered in 1832, by M. Pelletier, and meconine was discovered and well studied, says Magendie, about the same time, by M. Couerbe. They are both obtained from the ammoniacal solution from which morphia has been precipitated. Narceine is white and inodorous, and crystallizes in long needles; its taste is slightly bitter and almost metallic. It dissolves in 230 parts of boiling, or 375 parts of cold water. It melts at 92° of the centigrade thermometer (197° $\frac{3}{5}$ Fahr.) and is decomposed by a higher temperature. Its principal characters, however, are developed by the action of acids. When concentrated, they decompose it entirely, especially when aided by heat. When diluted with half their weight of water they combine with the narceine, and produce remarkable changes of colour. Thus, at the moment when the two bodies come in contact, a beautiful blue is produced; and if the water is absorbed by chloride of calcium, or by magnesia, a rose colour is produced. This singular property of narceine has induced M. Couerbe to call it the vegetable chameleon. Nitric acid changes it into oxalic acid.

*Meconine* is a white substance, crystallizing in six-sided prisms, two of the sides being larger than the others and parallel. It melts at 90° of the centigrade thermometer (194° Fahr.). At this temperature it is strongly acted upon by chlorine, which changes its colour to a fine blood-red, and turns it into a new acid, called the mechloic. M. Magendie has often injected narceine and meconine into the jugular vein of dogs, but without effect; and on man they have not yet been tried.

*Codeine* was discovered in 1832 by M. Robiquet. Finding that the muriate of morphia prepared according to Gregory's method did not yield a proper quantity of morphia, he suspected that it was impure. This turned out to be the case, and its impurity was occasioned by a new substance crystallizing in beautiful prisms, soluble in water, ether, and alcohol, and possessing very energetic alkaline properties. This was codeine.

M. Magendie has administered codeine to a considerable number of patients at the Hôtel-Dieu, and he finds that a single grain given at once or at twice is sufficient in some cases to produce sleep, which is not followed the next day by drowsiness or a sense of weight in the head, effects often produced by morphia. Several comparative experiments have seemed to show that a grain of codeine is equivalent to half a grain of pure morphia. Two grains of codeine have more than once excited nausea and even vomiting. Even when given in the dose of one grain, several patients requested that the medicine might be discontinued, as they said it made them sleep too much.

The muriate of morphia is more powerful than the uncombined alkali, and two grains cause not only sleep, but vertigo, nausea, and even vomiting. But under the use of this dose, M. Magendie has seen sciatica and facial neuralgia, after having resisted the long series of remedies employed against them, disappear as if by enchantment. M. Magendie has hitherto tried only the muriate and the nitrate of codeine, but he has found that several patients who had exhausted the narcotic power of morphia and its salts, still experienced the most satisfactory effects from the use of codeine, alternately with its nitrate or its muriate.

*Hydrocyanic ether* was discovered by M. Pelouze, a young chemist of great merit. Its effects are the same as those of prussic acid, but less in degree, and it may be administered in the same diseases, but its disagreeable smell is a strong obstacle to its general use. One patient, indeed, took a potion containing six drops of prussic ether, and experienced relief from a convulsive cough, without complaining of the odour; but, when administered at the Hôtel-Dieu, the patients were so disgusted by the smell, that our author was obliged to omit the medicine.

*Mannite.*—The manna of commerce, known by the name of manna in the tear, is treated with boiling alcohol, then filtered and allowed to crystallize; when cold the mannite is precipitated in small needles beautifully white. Mannite is soluble in water in almost any proportion, and between 105° and 110° of the centigrade thermometer (221° and 230° of Fahrenheit) it melts into a colourless liquid, which crystallizes on cooling: when heated more strongly, it burns and is decomposed like sugar. The dose for children is two drachms; half an ounce purges them too much.

*Lactic acid.*—We shall take our account of this substance from Dr. Gully's translation.

"*Process for procuring lactic acid.*—It is extracted both from milk and from the juice of beet-root. If from the latter, it should be left in a stove of a fixed temperature, between 25° and 30°. After a few days, viscous fermentation takes place, and hydrogen mixed with carburetted hydrogen is abundantly evolved. The fermentation ended, and the liquid restored to its former fluidity — which generally occupies two months, it is evaporated to a syrupy consistence; crystals of mannite then appear, and with them a sugar, having the properties of a grape sugar. The product of the evaporation is treated with alcohol, which dissolves the lactic acid, leaving a quantity of precipitated matters. The alcoholic extract is taken up by water, wherefrom a fresh deposit is made; the fluid is then saturated with carbonate of zinc, and a copious precipitation again takes place. Concentrated, the lactate of zinc crystallizes, is collected and heated with water, to which some animal charcoal previously washed with hydrochloric acid is added: the whole is then filtered, and the lactate of zinc, perfectly white, crystallizes: these crystals are again washed with boiling alcohol, in which they are insoluble. By successive treatment with baryta and sulphuric acid the lactic acid is separated and concentrated in vacuo. Finally on shaking it with sulphuric ether, which dissolves it, a flaky matter is separated. (See Ann. de Chimie et de Physique, April, 1833.)

"By a precisely similar process, milk affords lactic acid. M. Carriol has also discovered it in the aqueous solution of the strychnos nux vomica.

"*Physical and chemical properties of lactic acid.*—Concentrated in vacuo until it loses no more water, lactic acid is a colourless fluid of a syrupy consistence, and a density, at the temperature of 20°.5, equal to 1,215. It is inodorous, and excessively acid to the taste. It absorbs moisture from the atmosphere. Water and alcohol dissolve any quantity of it. It has the property of rapidly dissolving phosphate of lime, particularly that of the bones, a property worthy the attention of medical men.

*Mode of administering lactic acid.* — As lactic acid is the solvent of food in the stomach, I thought that it might be advantageously used in dyspepsia or simple debility of the digestive organs: and I have not been disappointed.

"I give it in the form of lemonade or lozenges.

*Lactic Lemonade.*

| | |
|---|---|
| Liquid lactic acid . . . . . | 1 to 4 gros. |
| Simple water . . . . . . | 1 pinte. |
| ——— syrup . . . . . . | 2 onces. |

*Lozenges of lactic acid.*

| | |
|---|---|
| Pure lactic acid . . . . . | 2 gros. |
| Powdered sugar . . . . | 1 once. |
| Tragacanth gum . . . . | q. s. |
| Volatile oil of vanilla . . . . | 4 drops. |

"The lozenges should weigh half a gros each, and be kept in a well-stopped glass. Six of them may be taken in twenty-four hours.

"From the facility with which lactic acid dissolves calcareous phosphate, it might be feasible to try it in cases of white or phosphate of lime gravel. I have not yet had an opportunity of doing so.

"I have commenced a series of clinical experiments with the lactates of soda, potass, &c. but the results are not yet ripe for publication."—(P. 193.)

Our readers will have observed in this extract a peculiarity of the translator: he does not translate the words *gros*, *once*, &c. but leaves the reader to find out their equivalent from a table at the end of the book. We are there told that the *gros*, a French weight, is 60 English grains; while a *gros*, a French measure, is one drachm and five minims in English measure, and at the same time that the French chemists always calculate fluids by weight. This is puzzling enough; but at p. 19 the directions given about a certain mixture are "Five gros (7 drachms and a half) to be taken morning and evening." On turning to the original p. 36, we find the direction to be "Pour une cuillerée à bouche matin et soir,"—i. e. a tablespoonful to be taken morning and evening. The translator says, indeed, at p. 211, that where he has mentioned 5 gros and a half a tablespoonful may be understood and administered, and he would have done far better to translate throughout *gros*, drachm, *once*, ounce, *cuillerée à bouche*, tablespoonful, *cuillerée à café*, teaspoonful, *livre*, pound, and so on.

The only other fault worth mentioning is the omission of several passages of the original, which, in a work of such remarkable succinctness as well as merit, is not commendable. Magendie (p. 149) speaks of *sulphate de quinine associé à l' émétique.* This is translated (p. 66) "combination with an emetic;" it should be "with tartar emetic."

In the following prescription there appears to be an error in the original, which is copied by Dr. Gully, at p. 125 of his translation:

"*Pommade d' hydrobromate de potasse bromurée.*

| | |
|---|---|
| Axonge pur . . . . . . . | 1 once. |
| Hydrobromate de potasse . . . | 24 grains. |
| Brôme liquide . . . . . . | 6 à 12 gros. |

"Mêlez. Par frictions."—(P. 261.)

It should probably be *grains* or *gouttes* instead of *gros.*

The translation, however, is on the whole a very good one, and those who cannot read the original with facility would do well to procure it.

The additional articles are on creosote, and the ioduret and hydriodate of iron. It is remarkable that the ergot of rye is omitted both by Magendie and his translator. Dr. Gully, writing in 1835, says that creosote was discovered last year, (it was discovered in 1832;) and asserts "that British practitioners have not essayed the effects of creosote," to which he makes an exception afterwards in favour of Dr. Copland.

There is an inconvenience attending the use of the ioduret and hydriodate of iron, which Dr. Gully has not mentioned; they are decomposed with extreme facility, and, (unlike most medicines, which lose their energy by decomposition,) the quantity of iodine disengaged becomes too large for a wholesome dose.

